# TDP-43 proteinopathy impairs mRNP granule mediated postsynaptic translation and mRNA metabolism

**DOI:** 10.7150/thno.51004

**Published:** 2021-01-01

**Authors:** Chia-En Wong, Lee-Way Jin, Yuan-Ping Chu, Wei-Yen Wei, Pei-Chuan Ho, Kuen-Jer Tsai

**Affiliations:** 1 Department of Medicine, College of Medicine, National Cheng Kung University, Tainan, Taiwan.; 2 Institute of Clinical Medicine, College of Medicine, National Cheng Kung University, Tainan, Taiwan.; 3 Department of Pathology and Laboratory Medicine, UC Davis Medical Center, California, USA.; 4 Research Center of Clinical Medicine, National Cheng Kung University Hospital, College of Medicine, National Cheng Kung University, Tainan, Taiwan.

**Keywords:** TDP-43, mRNP granule, postsynaptic, local translation, super-resolution microscopy

## Abstract

**Background:** Local protein synthesis and mRNA metabolism mediated by mRNP granules in the dendrites and the postsynaptic compartment is essential for synaptic remodeling and plasticity in neuronal cells. Dysregulation of these processes caused by TDP-43 proteinopathy leads to neurodegenerative diseases, such as frontotemporal lobar degeneration and amyotrophic lateral sclerosis.

**Methods:** Using biochemical analysis and imaging techniques, including super-resolution microscopy, we provide evidence, for the first time, for the postsynaptic localization of TDP-43 in mammalian synapses and we show that TDP-43 is a component of neuronal mRNP granules.

**Results:** With activity stimulation and various molecular approaches, we further demonstrate activity-dependent mRNP granule dynamics involving disassembly of mRNP granules, release of mRNAs, activation of local protein translation, and the impairment of granule disassembly in cellular, animal and human models of TDP-43 proteinopathy.

**Conclusion:** Our study elucidates the interplay between TDP-43 and neuronal mRNP granules in normal physiology and TDP-43 proteinopathy in the regulation of local protein translation and mRNA metabolism in the postsynaptic compartment.

## Introduction

Neurodegenerative diseases are a heterogeneous group of disorders characterized by progressive functional decline of the central nervous system accompanied by progressive loss of the structure and function of neurons in the brain and/or the spinal cord 1, 2. In neurodegenerative diseases including Alzheimer's disease, frontotemporal lobar degeneration (FTLD), amyotrophic lateral sclerosis (ALS) and Huntington's disease (HD), a common pathological feature is the appearance of abnormal protein aggregates 3-9. Previous studies have suggested that the pathogenic process of these abnormal protein aggregates involves abnormal protein folding and can result in loss-of-function of multiple nuclear and/or cytosolic proteins 10. In addition, previously published studies have proven that the formation of insoluble protein inclusions is also toxic, leading to impaired cellular function and neuronal death in the central nervous system 11-13. Taken together, the processes of abnormal protein folding and aggregation formation contribute both to the loss of normal physiological protein function and enhanced cellular toxicity, which ultimately lead to disease phenotypes.

RNA-binding proteins (RBPs) are involved extensively in various forms of neurodegenerative diseases 14, 15. RBPs are key components of messenger ribonucleoprotein particles (mRNPs) that associate and interact with mRNAs to form mRNA-protein complexes that participate in the regulation of sophisticated post-transcriptional gene regulation, including pre-mRNA splicing, RNA editing, mRNA transport and local translation 16. Recent studies have shown that mRNPs can be further assembled into mRNP granules, which are higher-order mRNA-protein complexes with cargo mRNAs associated with non-translating ribosomes. These translationally silenced mRNP granules include neuronal RNA transport granules, P bodies and stress granules 17, 18. There is evidence that cargo mRNAs in mRNP granules are translationally paused before completion of translation initiation and they traffic along axons and dendrites to presynaptic and postsynaptic terminals 19. Upon appropriate activation signals such as synaptic activity, translationally paused mRNP granules can be reactivated resulting in local translation in axons, dendrites and synapses 20-22. Among different RBPs, TAR DNA-binding protein 43 (TDP-43) is a ubiquitously expressed multifunctional DNA/RNA binding protein that is primarily nuclear, but shuttles between the cytoplasm and the nucleus 23. TDP-43 was identified as the major component of pathological cytoplasmic inclusions, which are the pathological hallmark of FTLD and ALS 24, 25. Previous studies have shown that both gain-of-function mutations and overexpression of TDP-43 are sufficient to cause FTLD and/or ALS phenotypes, highlighting the role of TDP-43 in disease pathogenesis 26-28. TDP-43 has thousands of mRNA targets, many of which play important role in central nervous system (CNS) development and synaptic homeostasis 23, 29. Despite its primary nuclear localization, TDP-43 isolated from both human and rodent brain cytosolic fractions binds to the 3' UTR of numerous target mRNAs 28. Additional studies have also suggested the important role of TDP-43 in mRNA transport and stability. For example, TDP-43 is involved in microtubule-dependent bidirectional axonal mRNA transport 30. Somatodendritic TDP-43 was also shown to regulate somatodendritic RNA metabolism 31. Together, these findings suggest a potential role for TDP-43 in neuronal plasticity by regulation of axodendritic local translation. Impairment of cytosolic TDP-43 function may be important for the pathogenesis of diseases featuring TDP-43 proteinopathy such as FTLD and ALS, yet direct evidence is lacking.

Here, we elucidated the interplay between TDP-43 and neuronal mRNP granules in TDP-43 proteinopathy. We provided evidence for the synaptic localization of cytosolic TDP-43 and for the first time directly visualized the postsynaptic localization of TDP-43 in mammalian synapses by far field super-resolution microscopy. We showed that TDP-43 was a component of neuronal mRNP granules and we delineated the neuronal activity-dependent granule dynamics in normal physiological conditions and in TDP-43 proteinopathy. Our work also provided insight into the involvement of neuronal mRNP granules in pathological TDP-43 inclusions in cellular and animal models, and FTLD-TDP patients.

## Results

### TDP-43 in the mouse CNS

First, we analyzed the distribution of TDP-43 in the mouse CNS using immunofluorescence. We detected TDP-43 in brain areas including the cortical layers and the hippocampus (Figure S1A). The sharp-line signals in the hippocampal area indicate a predominantly somatic localization of TDP-43. Co-staining of TDP-43 with the neuron marker NeuN and the nuclear marker DAPI revealed a predominantly nuclear localization of TDP-43, in agreement with previous studies 32, 33 (Figure S1B). Co-staining of TDP-43 and glial marker GFAP showed few colocalization, showing a predominant distribution of TDP-43 in neurons (Figure S1C).

### Subcellular localization of TDP-43

**Figure 1:**
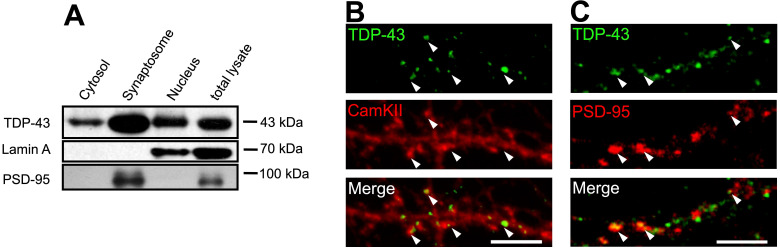
** Subcellular localization of TDP-43. (A)** Representative blot showing the different subcellular fractionation from the forebrain of adult mice. **(B-C)** Confocal microscopy images of TDP-43, CamKII or PSD-95 immunostaining in primary mouse cortical neuronal cells are shown. Arrowheads indicate the colocalization of TDP-43 with CamKII or PSD-95 puncta. Scale bar: 5 µm.

Next, we analyzed the subcellular localization of TDP-43. Subcellular fractionation from forebrain of adult mice were performed. Lamin A and PSD-95 were used as internal controls of nuclear and purified synaptosome fractions, respectively. Tissue fractionation showed that TDP-43 was detected in all fractions including nuclear, cytosolic and purified synaptosome fractions. This result suggests that in addition to its primary nuclear localization, TDP-43 also showed cytosolic localization and a potentially enriched synaptic localization (Figure 1A).

To further confirm the presence of axodendritic and synaptic TDP-43 molecules, we utilized confocal fluorescence microscope to identify the subcellular localization of TDP-43 in 19 day *in vitro* (19 DIV) primary mouse cortical neurons. We observed a predominant nuclear localization of TDP-43, while cytosolic TDP-43 puncta were also present. High magnification images showed that cytosolic TDP-43 puncta distributed along neurites and were colocalized with mushroom-like structures of dendritic spines labeled by CamKII (Figure 1B). Co-staining with the postsynaptic marker PSD-95 also showed colocalization of TDP-43 with PSD-95 puncta in neurites (Figure 1C). Together, these results suggest that cytosolic TDP-43 shows a synaptic subcellular localization in neurons.

### Super-resolution microscopy reveals postsynaptic localization of TDP-43 in dendritic spines

**Figure 2:**
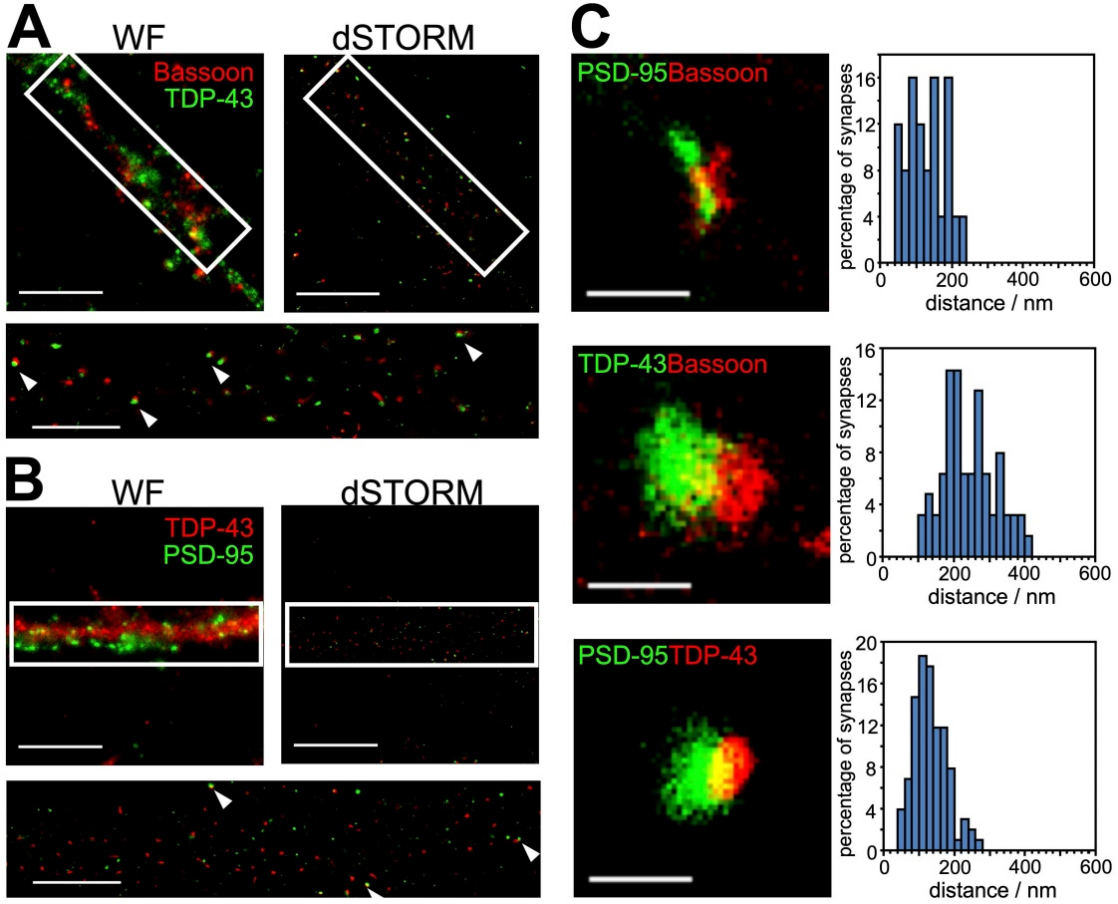
** dSTORM images and distance analysis revealed postsynaptic localization of synaptic TDP-43. (A)** Representative wide-field (WF) and dSTORM images of the presynaptic marker, Bassoon, and TDP-43 are shown. Arrowheads indicate the nearby colocalization of Bassoon and TDP-43 in magnified dSTORM imaging (white rectangle). **(B)** Representative wide-field and dSTORM images of TDP-43 and the postsynaptic marker PSD-95 are shown. Arrowheads indicate near-by colocalization of PSD-95 and TDP-43 in magnified dSTORM imaging (white rectangle). Scale bars: 10 µm and 5 µm (magnified dSTORM images). **(C)** Representative dSTORM images show the synaptic pairs between PSD-95, Bassoon and TDP-43 (left panels). Scale bar: 400 nm. Histograms showing the distribution of measured distance between PSD-95 and Bassoon, TDP-43 and Bassoon, PSD-95 and TDP-43 (right panels). The data represent the distance of n ≥ 25 synaptic pairs in n ≥ 3 biological repeats.

Hindered by diffraction limit, conventional fluorescence microscopy failed to resolve the exact localization of TDP-43 within synapses. In order to investigate the precise synaptic localization of TDP-43, we utilized super-resolution imaging with direct stochastic optical reconstruction microscopy (dSTORM). TDP-43 and reference synaptic proteins including the presynaptic and postsynaptic makers Bassoon and PSD-95 were immunolabeled in 19 DIV cortical neurons, which contain morphologically mature synapses. Wide-field immunofluorescence images showed puncta structure of TDP-43, Bassoon and PSD-95 distributed along neuronal processes. However, colocalization of TDP-43 with Bassoon and TDP-43 with PSD-95 was both observed in wide-field images (Figure 2A-B, left upper panel), thus we were unable to determine the exact synaptic localization of TDP-43. Next, we reconstructed dSTORM images (Figure 2A-B, right upper panel), which showed nearby colocalization of neighboring TDP-43 with Bassoon as well as TDP-43 with PSD-95 in the neuronal processes (Figure 2A-B, lower panel). In order to further determine whether TDP-43 is localized in presynaptic or postsynaptic areas, localization intensity profiles of the protein distributions along the presumed trans-synaptic axis were investigated to measure the trans-synaptic axial distance between TDP-43, Bassoon and PSD-95. The center of the mass for each cluster was determined by fitting to the localization intensity profiles with a simple Gaussian function and the distances between centers of mass (COMs) were measured along the trans-synaptic axis of the synapses.

The magnification in Figure 2C shows typical synapses of each combination of protein cluster pairs of Bassoon with PSD-95, Bassoon with TDP-43, and PSD-95 with TDP-43. Position of COMs were calculated according to the intensity profiles along the trans-synaptic axis and the distances between the respective protein pairs were measured (Figure S2) As previously described, mean distance derives from imaging a three dimensional structure in two dimensions; as a result, only 40% of all synapses with the highest distance were selected for synapses whose synaptic cleft is tilted out of and perpendicular to the imaging plane 34. More than 190 measurements were analyzed and the distances of all analyzed synapses were grouped in a histogram in 20 nm steps to display the distribution of all measurements (Figure 2C, right panels).

The analysis showed a mean distance of 245.1 ± 10.1 nm (mean ± SEM, n = 63) between TDP-43 and Bassoon, while the distance between TDP-43 and PSD-95 was 134.1 ± 5.0 nm (mean ± SEM, n = 102). These results indicate closer proximity of synaptic TDP-43 to the postsynaptic marker PSD-95 compared to the presynaptic marker Bassoon. In addition, the distance between Bassoon, and PSD-95 was also analyzed as control, showing a mean distance of 121.6 ± 9.5 nm (mean ± SEM, n = 25), which is in agreement with previously published measurements with super-resolution microscopy 35.

The distances between TDP-43 and presynaptic/postsynaptic markers imply two possible locations of TDP-43: 1) in the synaptic cleft and close to the postsynaptic membrane or 2) in the dendritic spine and distal to the postsynaptic density. Importantly, the Bassoon-TDP-43 distance was close to the sum of Bassoon-PSD-95 distance and PSD-95-TDP-43 distance, suggesting a postsynaptic localization of TDP-43 in dendritic spines. Taken together, by combining super-resolved dSTORM microscopy and distance analysis, these results revealed for the first time the postsynaptic localization of synaptic TDP-43 in the dendritic spines.

### TDP-43 is a component of neuronal RNA granules *in vitro* and *in vivo*

**Figure 3:**
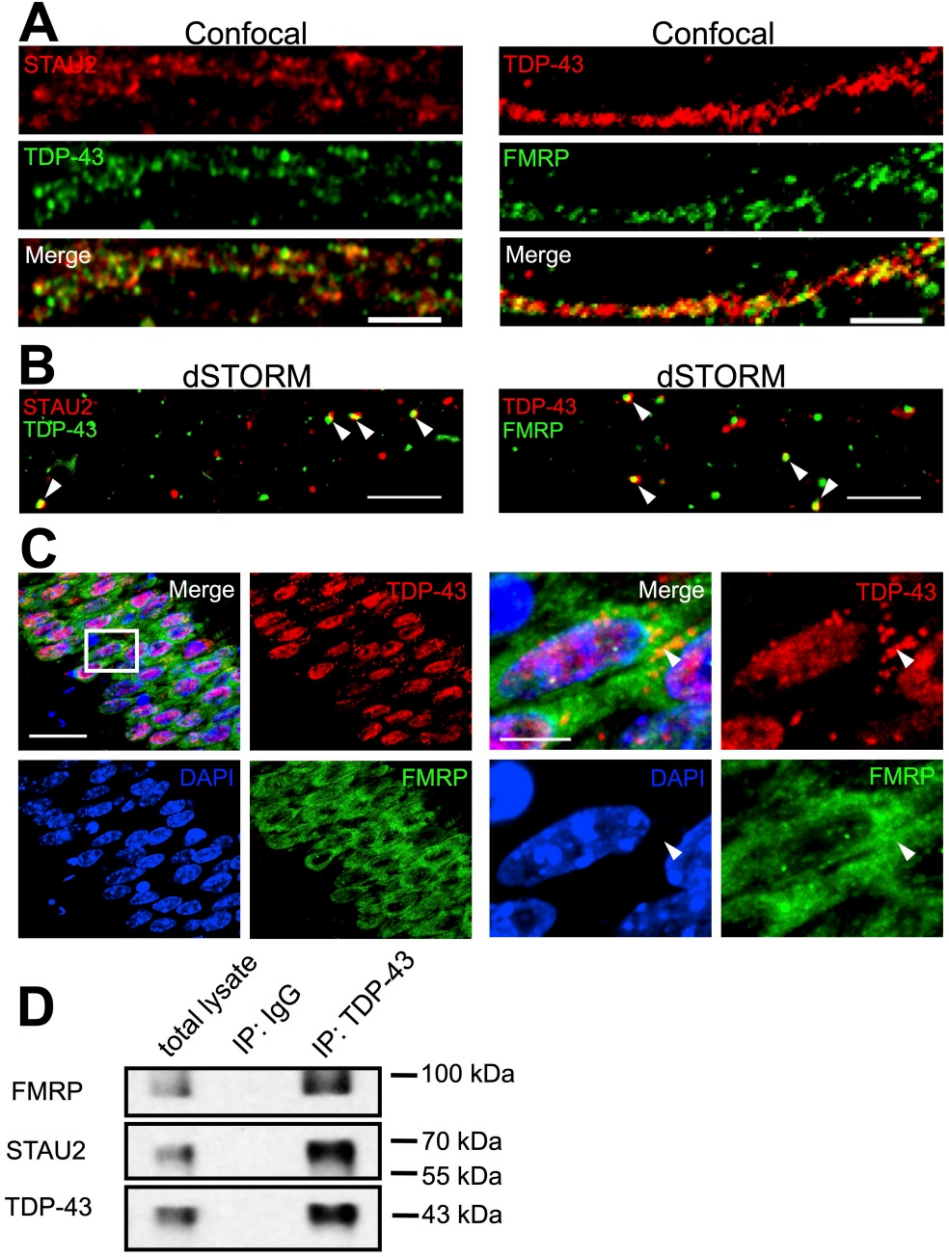
** TDP-43 is a component of neuronal RNA granules. (A)** Representative confocal microscopy images of TDP-43 with STAU2 or FMRP immunostaining in primary mouse cortical neuronal cells. Scale bar: 5 µm. **(B)** dSTORM images of TDP-43 with STAU2 or FMRP in primary mouse cortical neuronal cells. Arrowheads indicate the colocalized puncta of TDP-43 with STAU2 or FRMP. Scale bar: 5 µm. **(C)** Representative images showing immunofluorescence staining of TDP-43 and FMRP in adult *WT* mouse hippocampal CA1 area. Arrowheads indicate the cytosolic TDP-43 puncta. Scale bar: 20 µm and 5 µm (magnified images) **(D)** Representative blots showing the co-immunoprecipation of TDP-43 with FMRP and STAU2.

Cytosolic TDP-43 binds to numerous target mRNAs in the 3' UTR 23. Previous studies have shown the involvement of cytosolic TDP-43 in mRNA transport and stability 30, 36. Some studies have also proposed that TDP- 43 may be involved in the function of neuronal mRNP granules 37, 38. We asked whether cytosolic TDP-43 interacts with neuronal mRNP granule components and whether cytosolic TDP-43 is involved in the regulation of neuronal mRNP granule function and RNA metabolism. To address this, relations of TDP-43, Staufen 2 (STAU2) and Fragile X mental retardation protein (FMRP) was analyzed. STAU2 and FMRP are RNA binding proteins and were identified as key components and markers of neuronal mRNP granules 39. TDP-43, STAU2 and FMRP were immunostained in 19 DIV primary cortical neurons. Confocal imaging showed that both STAU2 and FMRP were distributed in puncta along the neurites, colocalized with TDP-43 (Figure 3A). To confirm colocalization of TDP-43 and neuronal granule markers, we utilized super-resolution microscopy. Reconstruction dSTORM images revealed true colocalization of TDP-43 with STAU2 puncta as well as TDP-43 with FMRP puncta (Figure 3B).

To investigate TDP-43* in vivo*, frozen sections from adult mice were prepared as described in Material and Method section followed by TDP-43, STAU2 and FMRP staining. Confocal microscopy showed a predominant nuclear localization of TDP-43 observed in the hippocampal CA1 area (Figure 3C, left panels). Additionally, cytosolic TDP-43 puncta were also present and colocalized with FMRP, as shown in magnified images (Figure 3C, right panels). In addition to immunofluorescence, biochemical analysis was also performed. Co-immunoprecipitation from whole brain lysates of adult mice revealed interactions between all three of TDP-43, STAU2 and FMRP (Figure 3D). Together, these results suggest that cytosolic TDP-43 interacts with neuronal mRNP granule markers and TDP-43 is a component of neuronal mRNP granules.

### Activity-dependent disassembly of TDP-43-containing neuronal RNA granules

mRNP granules are dynamic structures involved in local translation. Previous studies have shown that different mRNP granules including neuronal granules, processing bodies (PBs) and stress granules (SGs) interacted with each other and exchange components 39, 40. More importantly, in the cytoplasm, these translationally silenced mRNP granules undergo further reactivation, so that local translation can occur 20. The reactivation process involves release of paused mRNA-ribosome complexes from inhibitory RNA binding proteins resulting in the initiation of local translation upon certain signals including neuronal activity 41, 42.

**Figure 4:**
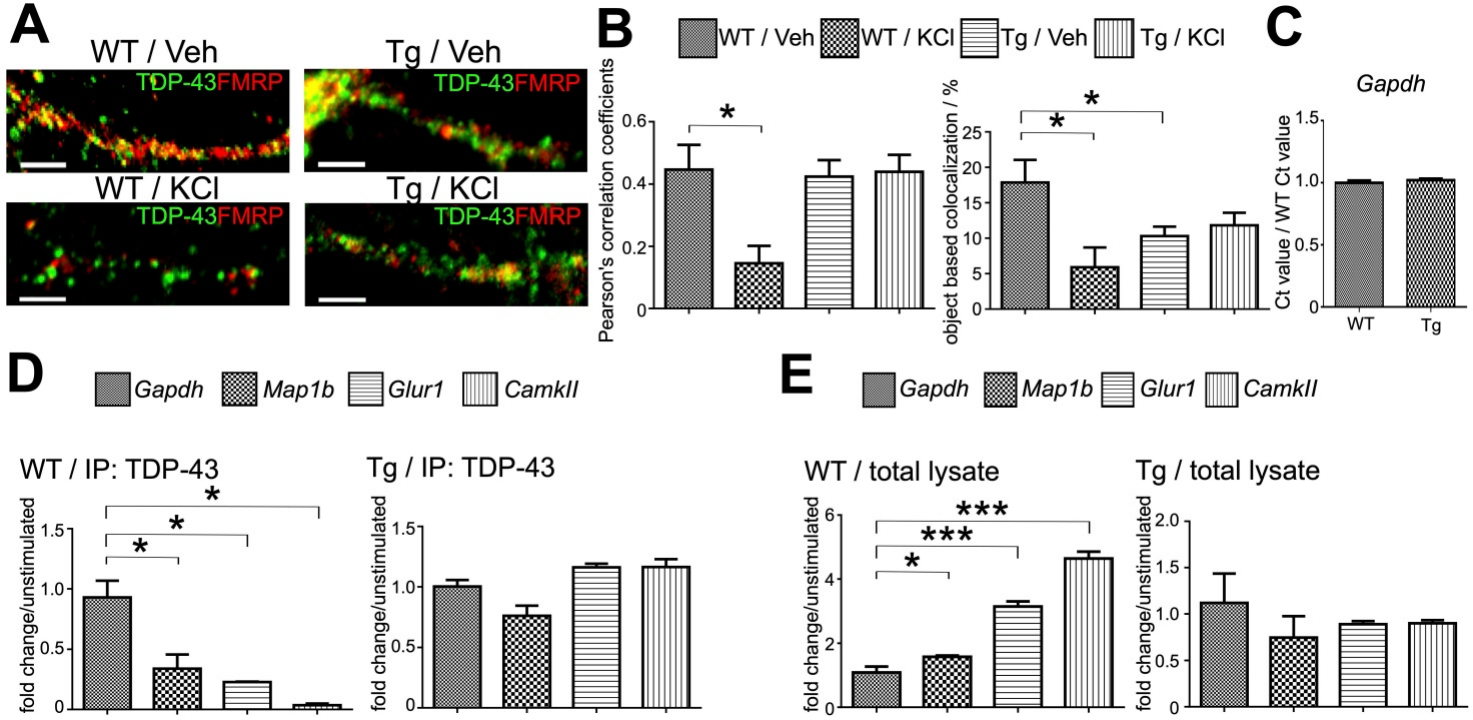
** Activity-dependent dynamic of TDP-43 containing neuronal RNA granules and its impairment in TDP-43 proteinopathy. (A)** Confocal microscopy images show the immunocytochemistry staining of TDP-43 and FMRP in *WT* or TDP-43 Tg primary mouse cortical neurons after KCl or vehicle treatment. Scale bar: 5 µm. **(B)** Pearson's correlation coefficient and object based colocalization analysis of TDP-43 and FMRP in *WT* or TDP-43 Tg neurons with or without KCl stimulation. The data represent the average and SEM of n ≥ 3 biological repeats. **(C)** Normalized *Gapdh* RNA levels in *WT* and TDP-43 Tg neurons. The data represent the average and SEM of n ≥ 3 biological repeats. **(D)** Representative bar graph showing the fold change of TDP-43 co-immunoprecipated RNA levels in KCl stimulated *WT* and TDP-43 Tg neurons compared to unstimulated neurons. The data represent the average and SEM of n ≥ 3 biological repeats. **(E)** Representative bar graph showing the fold change of free form of RNA levels in KCl-stimulated *WT* and TDP-43 Tg neurons compared to unstimulated neurons. The data represent the average and SEM of n ≥ 3 biological repeats.

Since the binding of TDP-43 to mRNAs inhibits translation, we hypothesized that upon reactivation by neuronal activity, TDP-43-containing mRNP granules disassemble and mRNP complexes unbind from TDP-43, thus, local translation can be completed. To test this hypothesis, we performed activity stimulation on 15 DIV primary mouse cortical neurons by repeated depolarization with 90 mM KCl three times. We analyzed the distribution of TDP-43 and FMRP in mouse cortical neurons with or without KCl stimulation with immunofluorescence. In comparison to unstimulated neurons, neurons treated with KCl showed lower number of colocalized puncta of TDP-43 with FMRP along the neurites (Figure 4A, left panels). Furthermore, confocal images of the unstimulated and stimulated neurites were analyzed by Pearson's correlation coefficient and object based colocalization analysis. A two-third reduction of colocalization of TDP-43 with FMRP was observed in both Pearson's correlation coefficient and object based colocalization analysis (Figure 4B). These results suggested that the interaction between TDP-43 and neuronal mRNP granules decreases upon KCl stimulation, supporting our hypothesis that TDP-43 containing mRNP granules disassemble and TDP-43 unbinds from mRNP complexes upon neuronal activity.

### Impairment of activity-dependent granule disassembly in TDP-43 proteinopathy

Because TDP-43 is involved extensively in neuronal mRNP granule metabolism and TDP-43 proteinopathy is sufficient to cause neurodegeneration and FTLD-related phenotypes, we asked whether TDP-43 proteinopathy may lead to impaired neuronal mRNP granule function. We used a TDP-43 overexpression transgenic mouse model (TDP-43 Tg) which mimics the FTLD phenotype including impaired learning/memory function and formation of cytosolic TDP-43 inclusions. The FTLD-TDP mouse model specifically overexpresses full-length TDP-43 in the forebrain under the control of the Ca^2+^/calmodulin-dependent kinase II (CaMKII) promoter 26.

We first investigated whether activity-dependent mRNP granule disassembly and TDP-43 unbinding were affected in TDP-43 proteinopathy. To this direction, 15 DIV primary mouse cortical neurons were prepared from P0 neonates from TDP-43 Tg mice and stimulated with repeated KCl depolarization. Unstimulated and stimulated TDP-43 Tg neurons were then analyzed with immuneofluorescence and confocal microscopy to investigate the distribution of TDP-43 and FMRP (Figure 4A, right panel). Our results showed that there were no obvious differences in the colocalization of TDP-43 with FMRP between unstimulated and KCl-treated TDP-43 Tg neurons. Further quantification with Pearson's correlation coefficient and object based colocalization confirmed that there was no difference in TDP-43-FMRP colocalization between stimulated and unstimulated TDP-43 Tg neurons (Figure 4B). These results suggest that activity-dependent mRNP granule disassembly was impaired in neurons with TDP-43 proteinopathy.

In order to investigate whether the activity-dependent mRNP granule disassembly and its impairment in TDP-43 proteinopathy are related to TDP-43-mRNA interactions and RNA metabolism, we investigated the profiles of TDP-43 target mRNAs in unstimulated and stimulated neurons. Previous studies have identified many mRNA targets for TDP-43 involved in the regulation of synaptic strength and neuronal plasticity 29, 43. For example, *Map1b*, *Glur1* and *CamkII* are involved in dendritic spine remodeling and regulation of synaptic signaling 44-46. Therefore, we investigated the amount of TPD-43 bound mRNAs and free form mRNAs. RNA-immunoprecipitation (RNA-IP) was performed from stimulated and unstimulated 15 DIV neuron lysates with anti-TDP-43 and RNA extracts were analyzed with quantitative PCR (qPCR) to detect mRNP granule-bound mRNAs. In addition, free form mRNA extracted from 15 DIV neuron lysates was also analyzed by qPCR. There was no difference in *Gapdh* mRNA levels between *WT* and TDP-43 Tg neurons (Figure 4C).

Immunoprecipitation with anti-TDP-43 from *WT* neurons stimulated by KCl showed decreased levels of TDP-43 bound target mRNAs with 0.34, 0.23 and 0.04-fold changes for *Map1b, Glur1* and *CamkII* mRNA levels, respectively, compared to unstimulated *WT* neurons, (Figure 4D, left panel). Importantly, the decrease of TDP-43 bound mRNAs after stimulation was observed with concurrent increase of free form *Map1b, Glur1* and *CamkII* mRNA levels with 1.58-, 3.14- and 4.64-fold change, respectively, compared to unstimulated *WT* neurons (Figure 4E, left panel). Together, these results supported the idea that the activity-dependent mRNP granule disassembly resulted in the release of mRNP bound mRNAs generating free form mRNAs.

However, the activity-dependent shift from mRNP bound mRNAs to free form mRNAs was not observed in TDP-43 proteinopathy. The level of anti-TDP-43 immunoprecipitated mRNAs and free form of RNAs from TDP-43 Tg neurons showed no difference for *Map1b, Glur1* and *CamkII* between unstimulated and stimulated neurons (Figure 4D-E, right panels). This result further suggests that the impairment of activity-dependent mRNP granule disassembly in TDP-43 proteinopathy resulted in subsequent impairment of mRNA release from TDP-43 containing mRNP granules.

### Impairment of mRNP granule-mediated local translation in TDP-43 proteinopathy

In order to determine whether the activity-dependent mRNP granule disassembly and subsequent mRNA release were linked to changes in local translation in the dendrites, we performed ribopuromycylation (RPM) assays to measure active translation in stimulated and unstimulated *WT* and TDP-43 Tg neurons.

**Figure 5:**
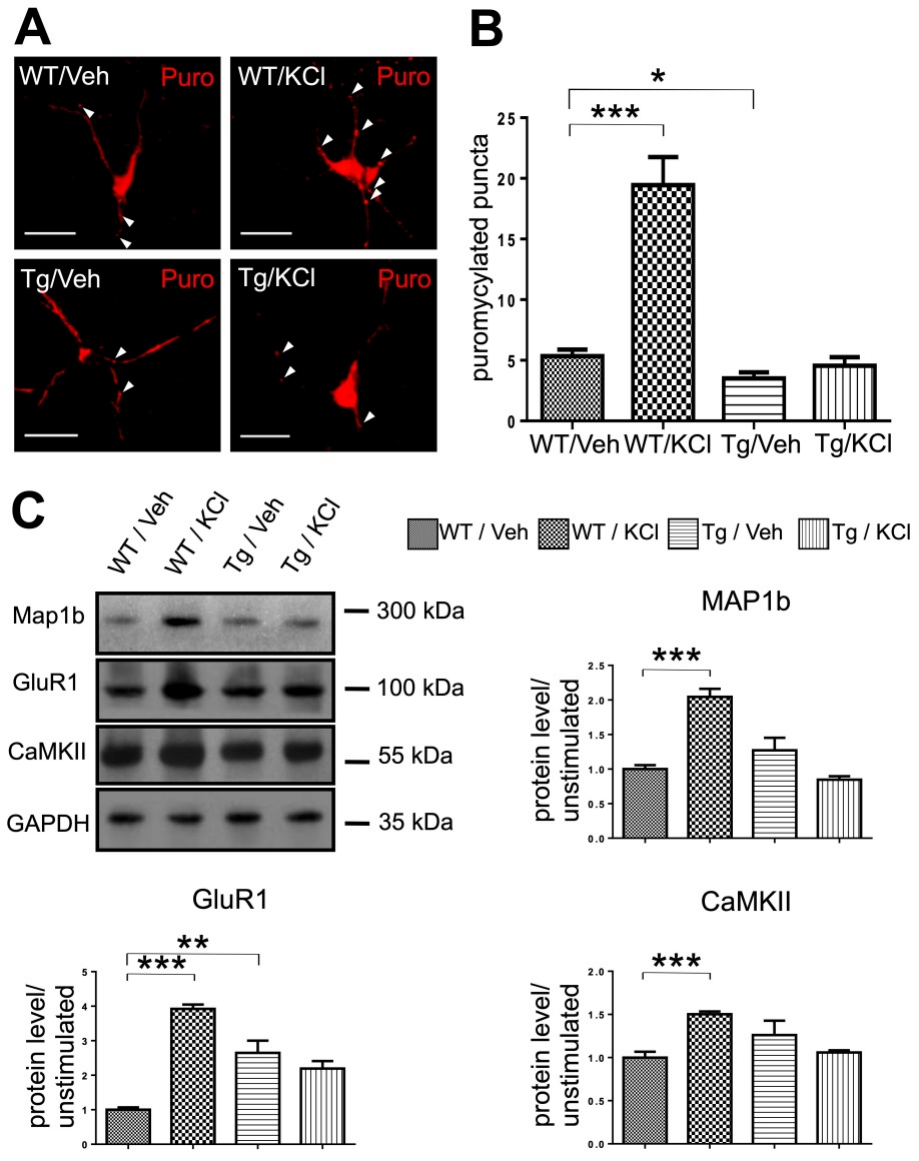
** mRNP granule mediated local translation is impaired in TDP-43 proteinopathy. (A)** Representative images showing the immunostaining of ribopuromycylated *WT* and TDP-43 Tg neurons with or without KCl treatment. Arrowheads indicate ribopuromycylated puncta. Scale bar: 20 µm. **(B)** Quantification of ribopuromycylated puncta per cell in *WT* and TDP-43 Tg neurons. The data represent the average and SEM of n ≥ 3 biological repeats. **(C)** Representative blots of MAP1b, GluR1 and CaMKII in *WT* and TDP-43 Tg neurons with or without KCl treatment are shown. The corresponding quantification of relative protein levels for MAP1b, GluR1 and CaMKII is also shown. The data represent the average and SEM of n ≥ 3 biological repeats.

In primary cultured neurons, puromycylated puncta were observed along the neurites (Figure 5A). In *WT* neurons, stimulation with KCl significantly increased the number of puromycin labeled puncta, suggesting an increase in local translation in the dendrites induced by neuronal activity (Figure 5B). Compared to *WT* neurons, TDP-43 Tg neurons showed a lower basal level of translation. Additionally, in TDP-43 Tg neurons, the activity-dependent increase of local translation was not observed and there was no difference in dendritic local translation between KCl-stimulated and unstimulated neurons baring TDP-43 proteinopathy (Figure 5B).

Furthermore, we analyzed the protein level of TDP-43 mRNA targets MAP1b, GluR1 and CaMKII in unstimulated and KCl-stimulated *WT* or Tg neurons. Our results showed that in *WT* neurons, KCl-stimulated neurons had 2.04-fold, 3.92-fold and 1.50-fold increase in relative protein levels of MAP1b, GluR1 and CaMKII, respectively, compared to unstimulated neurons. In contrast, there was no difference in the protein levels of TDP-43 target mRNAs in TDP-43 Tg neurons with or without stimulation (Figure 5C).

Together the RPM and western blot results suggest that granule mRNP disassembly and subsequent mRNA release were linked to increased local translation in dendrites and increased levels of TDP-43 target proteins. Importantly, these results further confirm our hypothesis that neuronal granule mediated RNA metabolism and local dendritic translation were impaired in a mouse model of TDP-43 proteinopathy. In summary, we reported that activity-dependent mRNP granule disassembly is accompanied by subsequent mRNA release, increase in local translation, increased levels of TDP-43 target proteins and their impairment in TDP-43 proteinopathy.

### Dysregulated RNA granules served as a component of pathological TDP-43 inclusions in the FTLD mouse model

**Figure 6:**
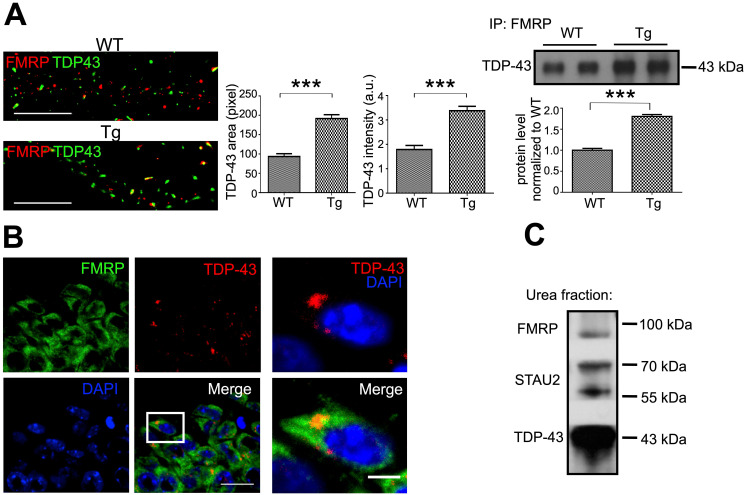
** Neuronal mRNP granules promote pathological TDP-43 inclusion. (A)** Representative dSTORM images of TDP-43 and FMRP in *WT* and TDP-43 Tg primary mouse cortical neuronal cells are shown (left panels). Scale bar: 5 µm. The corresponding quantification of TDP-43 area and TDP-43 intensity (middle panels) is shown. The data represent the average and SEM of n ≥ 3 biological repeats. Representative blots and corresponding quantification showing the immunoprecipated level of TDP-43 with FMRP in *WT* and TDP-43 Tg primary mouse cortical neuronal cells (right panels). **(B)** Representative immunofluorescence staining of TDP-43 and FMRP in adult TDP-43 Tg mouse hippocampal CA1 area is shown. Scale bar: 20 µm and 5 µm (magnified images) **(C)** Representative blot showing the expression of TDP-43, STAU2 and FMRP in urea soluble fractions.

We characterized the metabolism of TDP-43 containing mRNP granules; however, the role of neuronal mRNP granules in pathological TDP-43 inclusions remained elusive. To further clarify the role of neuronal mRNP granules in pathological TDP-43 inclusions, we investigated whether mRNP granule markers were present in TDP-43 pathological inclusions. Co-immunoprecipitation with anti-TDP-43 showed interactions between TDP-43 and the neuronal mRNP granule markers FMRP and STAU2 (Figure 3D). Next, to directly visualize the relation between TDP-43 and neuronal mRNP granule markers in *WT* and TDP-43 proteinopathy, dSTORM images of immunolabeled TDP-43 and FMRP were examined. Our results showed that TDP-43 containing neuronal mRNP granules colocalized in puncta with FMRP (Figure 6A, left panel). Quantification revealed increased TDP-43 positive area and increased TDP-43 total intensity per FMRP/TDP-43 colocalized puncta in TDP-43 Tg compared to *WT* neurons (Figure 6A, middle panels), which suggested increased relative amount of TDP-43 interacting with FMRP, in agreement with our co-immunoprecipitation results (Figure 6A, right panel).

Moreover, to investigate the role of neuronal mRNP granules in pathological TDP-43 inclusions *in vivo*, frozen sections from one-year-old FTLD mouse brains featuring TDP-43 proteinopathy were analyzed with immunofluorescence. In contrast to the predominant nuclear localization of TDP-43 in *WT* mouse brain sections (Figure 3C), immunofluorescence imaging analysis of TDP-43 Tg mouse brains showed predominant cytosolic TDP-43 puncta distribution in hippocampal CA1 neurons. There was little colocalization between TDP-43 and DAPI, indicating that cytosolic puncta distribution was accompanied by nuclear clearance of TDP-43 (Figure 6B, left panel). Additionally, colocalization of TDP-43 and FMRP was also observed in magnified images (Figure 6B, right panel).

Next, we performed urea fractionation to purify urea-soluble TDP-43 inclusions from 1-year-old FTLD mouse brains. Urea fractionation was analyzed by western blot, which showed that the neuronal mRNP granule markers STAU2 and FMRP were co-purified within the urea-soluble TDP-43 fraction indicating that neuronal mRNP granule components were present in pathological TDP-43 inclusions (Figure 6C).

### Involvement of neuronal mRNP granules in the formation of TDP-43 cytosolic inclusions

**Figure 7:**
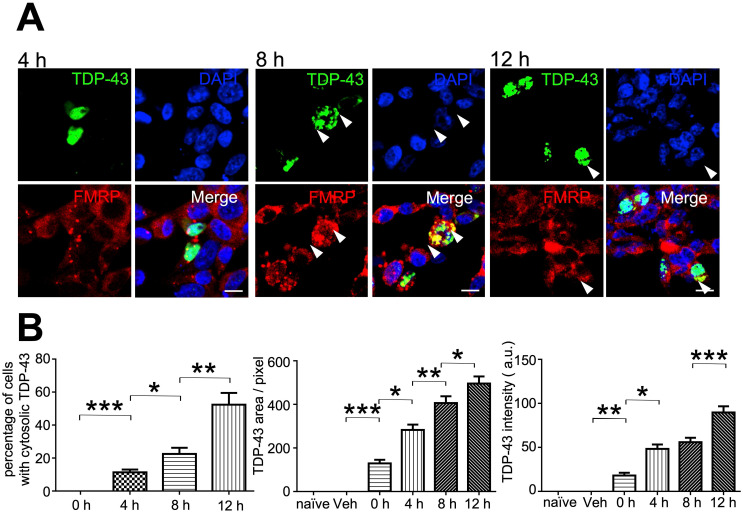
** mRNP granules participate in the formation of TDP-43 cytosolic inclusions. (A)** Representative immunocytochemistry staining of FMRP in GFP-TDP-43 transfected HEK293T cells after TDP-43 overexpression for 4, 8 and 12 h is shown. Arrowheads indicate cytosolic TDP-43 puncta. Scale bar: 20 µm. (**B**) Bar graph showing the percentage of cells with cytosolic TDP-43 (left panel) and quantification of TDP-43 area and intensity per transfected cell (middle and right panels). The data represent the average and SEM of n ≥ 3 biological repeats.

Since our evidence had suggested that neuronal mRNP granules were involved in pathological TDP-43 inclusions, we further investigated whether mRNP granules were present during the formation of pathological TDP-43 inclusions. Here, we used a cellular model to study the formation of TDP-43 inclusions. TDP-43-GFP was transfected and overexpressed in HEK293T cells. After transfection for 24 h, the protease inhibitor MG-132 was added to the cell culture to allow overexpression and formation of cytosolic TDP-43 inclusions. HEK293T cells were observed under confocal microscopy at 0, 4, 8 and 12 h after MG-132 treatment. As controls group, we used un-transfected or vehicle-transfected HEK293T cells, which showed no TDP-43-GFP signal (Figure S3, left panels). On the other hand, TDP-43-GFP transfected cells showed positive TDP-43 signal at all time-points after MG-132 treatment (Figure S3, right panels). Cytosolic TDP-43 was not present in HEK293T cells but observed after MG-132 treatment and the percentage of cells with cytosolic TDP-43 signal among all transfected cells (defined as cells with TDP-43 positive signal, regardless of subcellular localization) was increased in a time-dependent manner (Figure 7B, left panel).

In addition, TDP-43 inclusion bodies presenting as dense cytosolic puncta were observed in HEK293T cells after 8 and 12 h MG-132 treatment (Figure 7A, middle and right panels). We further analyzed the average TDP-43 area and total TDP-43 intensity per transfected cell. The analysis showed an increase in TDP-43 area and total TDP-43 intensity per transfected cell in a time-dependent manner, which revealed the accumulation of cytosolic TDP-43 over time during inclusion body formation (Figure 7B, middle and right panels). Importantly, we also observed colocalization between the mRNP granule marker FRMP and TDP-43 in all time-points in the presence of cytosolic TDP-43 4, 8 and 12 h after treatment (Figure 7A), further suggesting the involvement of mRNP granule components during accumulation of cytosolic TDP-43 over time. Taken together, these results suggest not only that mRNP granules were present in TDP-43 pathological inclusions, but were also involved in TDP-43 inclusion formation.

### Role of neuronal mRNP granule in pathological TDP-43 inclusions in the brain of FTLD-TDP patients

Our evidence indicated a role for neuronal mRNP granules in pathological TDP-43 inclusions. However, these experiments were performed in animal or cellular models. We wonder whether similar evidence could be observed in clinically relevant models.

**Figure 8:**
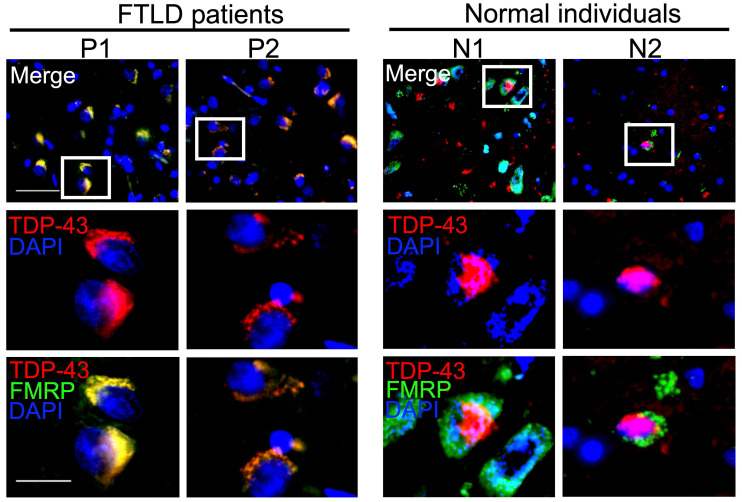
** Neuronal mRNP granules are present in TDP-43 proteinopathies of FTLD-TDP patients.** Representative immunofluorescence staining showing TDP-43 and FMRP expression in the frontal cortex of FTLD patients (left panels) and healthy individuals (right panels).

We investigated cerebral cortical sections obtained from healthy individuals and FTLD patients with TDP-43 proteinopathies. In healthy individuals, TDP-43 showed a predominantly nuclear pattern and FMRP presented as cytosolic puncta in the cerebral cortex (Figure 8, right panels). In contrast, FTLD-TDP pathological hallmarks with dense cytosolic TDP-43 aggregations were accompanied by clearance of nuclear TDP-43, in the cerebral cortex of FTLD-TDP patients (Figure 8, left panels). Importantly, counterstaining with FMRP showed a high degree of colocalization between FMRP and cytosolic TDP-43 aggregates in the cerebral cortex of all FTLD-TDP patients examined (Figure 8 and Figure S4). These results further supported the involvement of neuronal mRNP granules in TDP-43 proteinopathy and pathological TDP-43 inclusions in FTLD-TDP patients, consistent with the findings from animal and cellular experiments.

## Discussion

A detailed characterization of the interplay between TDP-43 and neuronal mRNP granules in TDP-43 proteinopathy will increase our understanding of the molecular pathogenic mechanisms of FTLD and of the metabolism of neuronal mRNP granules. Our work provided several important insights into these issues.

First, the biochemical fractionation and immunofluorescence experiments determined that besides the predominant nuclear localization, cytosolic localization of TDP-43 in neurons was also observed, consistent with previous reports 47, 48. Next, super-resolution imaging and other advance techniques can be utilized to provide detailed subcellular information or consequently therapeutic potential 49-52. Further investigation using super-resolved dSTORM microscopy revealed close proximity with nearby colocalization of neighboring TDP-43 with pre/postsynaptic markers. In addition, distance analysis here provided evidence for the first time for the postsynaptic localization of synaptic TDP-43 in the dendritic spines. Since TDP-43 is an RNA/DNA binding protein and serves as a translation repressor 53, its localization in the dendritic spines may suggest a potential role for TDP-43 in the regulation of synaptic local RNA metabolism.

In agreement with previous studies suggesting the involvement of TDP-43 in neuronal mRNP granules 30, 54, we reported that TDP-43 is a neuronal mRNP granule component and revealed its activity-dependent dynamics. In the present study, neuronal activity was simulated by a protocol of repetitive KCl stimulation adopted from the literature with minor modifications 55, 56. A total of three stimulations were performed, each involving a treatment of 90 mM KCl for three minutes. Previous studies reported that 90 mM KCl brings the neurons' membrane potential close to 0 mV, and provides maximal voltage-dependent calcium influx 55. Importantly, between each stimulation, a spaced recovery for 10 minutes was performed by replacing the high KCl concentration medium to the original culture medium. The 10-minute-recovery prevented the neurons from prolonged exposure to depolarizing medium and thus decreased the excitatory toxicity. Additionally, the stimulated neurons were harvested 30 minutes after last stimulation. The 30-minute-window offers time for any activity-dependent plasticity to occur.

Importantly, our observations suggest a potential mechanism of TDP-43 in the regulation of local synaptic translation. We showed the dynamics of TDP-43 containing neuronal mRNP granules: upon neuronal activity, mRNP granules disassembled and downstream local translation was induced after unbinding and release of TDP-43 target mRNAs. These downstream mRNA targets, including *Map1b, Glur1* and *CamkII* play critical roles in synapse remodeling 46, 57, 58, also highlighting the crucial role of TDP-43 regulation in synaptic plasticity.

The disassembly of mRNP granules can be facilitated by various mechanisms including exiting of mRNPs, decay of scaffold mRNAs, proteasomal turnover of scaffold proteins, and chaperon-mediated disassembly 17. Previous reports have addressed the effect of neuronal activity on TDP-43, showing that depolarization of rat hippocampal neurons regulated TDP-43 migration in the dendrites 37. It is probable that the activity-dependent TDP-43 migration results in the exiting of mRNPs and further leads to mRNP granule disassembly. Additionally, proteasome-dependent protein degradation was regulated by neuronal activity 59. It is also possible that mRNP granule disassembly could be triggered by activity-dependent proteasome degradation. However, the exact mechanism remains unclear and needs to be confirmed by experiments in the future.

On the other hand, our work revealed that TDP-43 proteinopathy perturbed the activity-dependent dynamics of TDP-43 mRNP granules and resulted in impaired local translation as well as impaired RNA metabolism of plasticity related mRNAs.

In addition, we reported excessive accumulation of TDP-43 in neuronal mRNP granules. Since previous studies have shown that intermolecular interactions of TDP-43 via C-terminal low complexity domain resulted in tight binding potentially causing formation of pathological inclusions 60, 61, it is probable that tight binding of neuronal mRNP granule components resulted from excessive accumulation of TDP-43 may hinder the disassembly of neuronal mRNP granules and impair subsequent mRNA release and local translation. Our results revealed the potential mechanism of TDP-43 proteinopathy related impairment of synaptic plasticity, which resulted in impaired learning and memory in FTLD 26, 62.

Given the accumulation of TDP-43 in neuronal mRNP granules, it is also probable that mRNP granules with TDP-43 accumulation may serve as seeds for TDP-43 aggregation and eventually lead to formation of pathological inclusions. This was supported by our results showing the presence of mRNP granule components both in TDP-43 pathological inclusions and during the process of inclusion formation as well. Importantly, we also confirmed the pathological phenotype in the brain of FTLD-TDP patients.

Additionally, comparison between unstimulated *WT* and TDP-43 Tg neurons revealed a difference in basal protein level of GluR1, indicating an increased amount of glutamate AMPA receptor in TDP-43 Tg neurons. This result may imply the potential disruption of glutamate receptors caused by TDP-43 proteinopathy, where the functional synaptic deficit in excitatory neurons was compensated by up-regulation of AMPA receptors. This is consistent with previous studies showing disruption of glutamate receptors in models of neurodegeneration 63, 64.

Taken together, these results suggest a model of FTLD pathogenesis, in which TDP-43 dysregulation in postsynaptic density first caused perturbation of normal neuronal mRNP granule function and led to impairment of local translation dependent synaptic plasticity and decline of learning memory function. At a later stage of disease, further accumulation of TDP-43 in mRNP granules served as seeds during the formation of pathological inclusions, which exert neurotoxicity leading to neuronal death 65, 66.

Looking forward, it will be important to further investigate the exact molecular mechanism of TDP-43 function in the regulation of activity-dependent neuronal mRNP granule dynamics in normal and pathological conditions. Understanding the basis of these processes may provide potential therapeutic strategies to synaptic dysfunction in early stage FTLD. In-depth knowledge about pathological inclusion formation may reveal further therapeutic targets to prevent or reverse the formation of TDP-43 pathological inclusions.

The observations reported here are likely to pertain to TDP-43 related neurodegenerative diseases including FTLD and ALS. In summary, our work confirmed the cytosolic and dendritic localization of TDP-43, but also showed an additional and precise localization of TDP-43 at the postsynaptic density in primary mouse cortical neurons. We demonstrated the involvement of TDP-43 as a component of neuronal mRNP granules and in the regulation of activity-dependent granule dynamics and local dendritic translation. In cellular, animal and human models of TDP-43 proteinopathy, impairment of neuronal mRNP granule function and involvement of neuronal mRNP granules in the formation of pathological inclusions was also shown. Our work provided new insights into the pathogenesis and potential therapeutic targets of FTLD.

## Materials and Methods

### Animal models

All experiments were in accordance with the guidelines of the Institute of Animal Use and Care Committee at National Cheng Kung University (NCKU), Taiwan. Mice were bred at the NCKU laboratory animal center. Water and food were provided *ad libitum*. The FTLD-TDP transgenic mouse model expressed the full-length mouse TDP-43 cDNA under the control of an 8.5-kb calcium/calmodulin-dependent protein kinase II (CaMKII) promoter and overexpressed TDP-43 in the forebrain. PCR genotyping was performed to identify whether the mice were transgene-positive. Six months old FTLD-TDP transgenic mice showed cytosolic TDP-43 aggregation accompanied by nuclear TDP-43 depletion in the brain, which mimics the hallmarks of TDP-43 proteinopathy in the brains of FTLD-TDP patients 26.

### Sub-cellular fractionation

Sub-fractionation of mouse brain tissue was performed as described previously with some modifications 67. Briefly, grossly dissected forebrain tissue was homogenized in buffer (0.32 M sucrose, 20 mM HEPES, pH 7.4, with protease inhibitors) and centrifuged at 1000 × g for 10 min to pellet membrane fragments and nuclei. The cytosolic fraction contained in the supernatant was collected, which was then centrifuged at 17,000 × g for 15 min to obtain the pellet containing the crude synaptosome fraction, which are synaptosomes together with mitochondria and microsomes. This crude synaptosome fraction was further purified using a discontinuous sucrose density gradient consisting of a 0.8 M sucrose layer on top of a 1.2 M sucrose layer. The crude synaptosome suspension was layered on top of the 0.8 M sucrose layer and centrifuged at 54,000 × g for 90 min. The purified synaptosomal fraction was obtained from the interface between 0.8 M sucrose and 1.2 M sucrose.

### Protein extraction

For protein extraction, mice were sacrificed with rapid cervical dislocation and the cortex was isolated. Forebrains containing cortex were resuspended in ice-cold RIPA lysis buffer (50 mM Tris-HCl, pH 8.0, 150 mM NaCl, 1% NP40, 0.5% sodium deoxycholate, 0.1% SDS) containing a protease inhibitor cocktail tablet (Roche) and the mixture of phosphatase inhibitor cocktail 2 and 3 (Sigma-Aldrich). Homogenates were centrifuged and supernatants were collected as total cell lysates.

### Western blot analysis

Proteins extracts (15-30 μg), quantified with the Bradford protein assay (Bio-rad), were resuspended in loading buffer and subjected to polyacrylamide gel electrophoresis followed by transfer to nitrocellulose membrane (Whatman). After blocking, membranes were incubated overnight at 4℃ with primary antibodies at optimal dilution (1:2000 for TDP-43 from Proteintech Cat# 10782-2 AP, and Lamin A from Sigma Cat# L1293; 1:1000 for PSD-95 from Abcam Cat# ab2723, FMRP from Cell Signaling Technology Cat# 4317, and STAU2 from Abcam Cat# 184009). Then, membranes were incubated with horseradish peroxidase-linked (HRP)-conjugated secondary antibodies (PerkinElmer) and western blot analysis was performed using Western Lightning^®^ Plus-ECL (PerkinElmer).

### Primary neuronal culture and drug treatment

Before culture, 20-mm glass coverslips were coated with 1 mg/mL poly-D-lysine at room temperature for 20 minutes; coated coverslips were then placed into 12-well culture plates. Mouse neonates at postnatal day 0 were sacrificed and their cerebral cortices were excised under a dissection microscope. Cortical tissues were triturated for disaggregation and plated on poly-D-lysine-coated glass coverslips in Neurobasal medium containing B27 serum-free supplement and antibiotics (50 U/mL penicillin and 50 μg/mL streptomycin). Cultures were incubated at 37°C in an 95% air, 5% CO_2_, and 90% relative humidity. Half of the growth medium was replaced every two days. Primary neurons were cultured for 15 to 19 days prior to immunostaining. For repetitive stimulation with KCl 55, neurons (15 days *in vitro*) were treated repetitively with 90 mM KCl for 3 min and a spaced recovery for 10 min. Neuronal cells were harvested or fixed 30 min after the last stimulation.

### Immunofluorescence staining

For immunohistochemistry of the mouse brains, mice were anesthetized and transcardial perfusion was performed with 4% paraformaldehyde (PFA) in PBS. The brain was isolated and embedded in paraffin blocks. Paraffin sections were generated and brains were sectioned at 10-μm-thick for immunofluorescence. Sections were deparaffinized in xylene and covered with citrate buffer for antigen retrieval. Subsequently, sections were permeabilized with 0.5% Triton X-100 (Sigma-Aldrich) and blocked with 5% bovine serum albumin for 1 h at room temperature. After blocking, sections were incubated overnight at 4°C with primary antibodies (1:300 for anti-FMRP from Cell Signaling Technology Cat# 4317, anti-TDP-43 from Proteintech Cat# 10782-2 AP, anti-NeuN from Millipore Cat# MAB377, and anti-GFAP from Millipore Cat# MAB3402), followed by incubation with secondary antibodies (Alexa Fluor® 594 and Alexa Fluor® 488) for 1 h. Nuclei were counterstained with DAPI (1:600, Sigma-Aldrich). Coverslips were mounted with fluorescent mounting medium.

For immunocytochemistry, cells were washed with PBS and fixed with 4% PFA for 15 min. Cells were then permeabilized with 0.1% Triton X-100 and blocked with 5% bovine serum albumin for 1 h at room temperature. After blocking, sections were incubated overnight at 4°C with primary antibodies (anti-CaMKII from Chemicon Cat# MAB3119, anti-PSD-95 from Abcam Cat# ab2723, anti-Bassoon from Synaptic Systems Cat# 141003, anti-TDP-43 from Proteintech Cat# 10782-2 AP, anti-FMRP from Cell Signaling Technology Cat# 4317, and anti-STAU2 from Abcam Cat #184009), followed by incubation with secondary antibodies for 1 h. Nuclei were counterstained with DAPI (1:5000). Images were acquired using an upright fluorescence microscope (BX51; Olympus) with 20X and 40X objective lens and a laser Scanning Confocal Microscope (C1-Si; Nikon) with 40X objective lens.

### dSTORM microscopy imaging buffer preparation

We used the imaging buffer composition suggested in a previously reported protocol 68. Buffer A was composed of 10 mM TRIS (pH 8.0) and 50 mM NaCl, while buffer B was composed of 50 mM TRIS (pH 8.0), 10 mM NaCl, and 10% glucose. GLOX solution (1 mL) was prepared by vortex mixing a solution of 56 mg glucose oxidase, 200 μL catalase (17 mg/mL), and 800 μL buffer A. MEA solution (1 M, 1 mL) was prepared using 77 mg MEA and 1 mL 0.25 N HCl. In each chamber of the 20-mm glass coverslips, 500 μL imaging buffer were added by mixing 5 μL GLOX solution, 50 μL MEA solution, and 445 μL buffer B on ice. After filling the chamber, it was covered immediately to avoid replenishment of the dissolved oxygen.

### dSTORM imaging

Alexa Fluor® 647 and Alexa Fluor® 568 secondary antibodies were used for dSTORM imaging. The microscope was constructed around an Olympus IX-83 automated inverted microscope. For illumination, an objective-type, total internal reflection fluorescence, oil-immersion objective (APON 60×; NA 1.49 total internal reflection fluorescence; Olympus) was used. A multiline laser source (405, 488, 561, and 640 nm; Andor Technology) was used for excitation and activation. Single-molecular localization signals were separated using appropriate filters (Andor Technology) and detected using electron-multiplying charge-coupled device camera (iXon Ultra 897; Andor Technology). Before dynamic image movie acquisition, conventional fluorescence images were acquired to determine the region of interest. Specifically, 10,000 frame image series were recorded with an exposure time of 50 ms (20 frames per second). The acquired image series were then analyzed using MetaMorph® Super-Resolution System (Molecular Devices) to generate reconstructed dSTORM images.

### Co-immunoprecipitation and RNA-immunoprecipitation

Cells/forebrain tissue were lysed in RIPA lysis buffer (50 mM Tris-HCl, pH 8.0, 150 mM NaCl, 1% NP40, 0.5% sodium deoxycholate, 0.1% SDS) containing a protease inhibitor cocktail tablet (Roche). Extracts were quantified with the Bradford protein assay (Bio-rad). Protein extracts (0.5 mg-1 mg) were diluted to 0.5 mg/ml with lysis buffer.

Co-immunoprecipitation was performed using the Catch and Release v2.0 Reversible Immunoprecipitation System (Millipore) according to the manufacturer's instructions with either 4 μg anti-TDP-43 (Proteintech, Cat# 10782-2 AP) or 4 μg of anti-FMRP (Cell Signaling Technology, Cat# 4317). For western blot analysis, precipitated proteins were washed and subsequently eluted in denaturation buffer and heated at 94 °C for 3 min. For RNA-immunoprecipitation analysis, precipitated proteins were washed and subsequently eluted in non-denaturation buffer, and RNAs in the immunoprecipitates were purified using Quick-RNA™ Microprep Kit (ZYMO RESEARCH) according to the manufacturer's instructions.

### Quantitative RT-PCR

For quantitative RT-PCR analysis, first strand cDNA synthesis was performed using Superscript RT (Invitrogen). Real time PCR was performed using the Power SYBR Green PCR Master Mix (Applied Biosystems) and specific primers for 25-33 cycles of 30 s at 94 °C, 30 s at 56 °C, and 1 min at 72 °C. The following primers were used: *Map1b*, 5'-TGGGACACAAACCTGATTGA-3' and 5'- TGAAAATCTCTATGAAGTTCT-3'; *GluR1*, 5'-CTAGGCTGCCTGAACCTTTG-3' and 5'-GGGAAGATTGAATGGAAGCA-3'; *CamKII*, 5'-AAAGTGCGCAAACAGGAAAT-3' and 5'-AGGTGGATGTGAGGGTTCAG-3'. *Gapdh* mRNA was used as internal control to normalize RNA levels.

### Ribopuromycylation assay

Ribopuromycylation was performed as described previously with some modifications 69, 70. Briefly, primary neuronal cells on coverslips were incubated with Neurobasal medium containing B27 serum-free supplement with 1 mg/ml puromycin and 100 μg/ml cycloheximide for 5 min at 37 °C. Cells were then washed with Neurobasal medium containing B27 serum-free supplement and fixed for 15 min with 4% PFA in PBS. For immunofluorescence, cells were permeabilized with 0.1% Triton X-100 and blocked with 5% bovine serum albumin for 1 h at room temperature. After blocking, sections were incubated overnight at 4 ℃ with anti-puromycin antibodies (1: 300, Millipore, Cat# MABE343), followed by incubation with secondary antibodies for 1 h.

### Urea-soluble fraction preparation

For analysis of urea-soluble proteins, forebrain tissues were dissected and extracted with serial buffers with increasing reducing power as previously described. Briefly, forebrains were extracted sequentially at 5 mL/g (volume/weight) with low-salt (LS) buffer (10 mM Tris, pH 7.5, 5 mM EDTA, 1 mM DTT, 10% sucrose containing protease inhibitors), high-salt Triton X-100 (TX) buffer [LS buffer + 1% Triton X-100 + 0.5 M NaCl], myelin flotation buffer [TX buffer containing 30% sucrose], and sarkosyl (SARK) buffer [LS + 1% N-lauroyl-sarcosine + 0.5 M NaCl]. The SARK-insoluble fractions were further extracted in 0.25 mL/g urea buffer (7 M urea, 2 M thiourea, 4% 3-[(3-Cholamidopropyl)dimethylammonio]-1-propanesulfonate, 30 mM Tris, pH 8.5). The urea-soluble proteins were then subjected to Western blot analysis.

### HEK 293T cell culture and TDP-43 overexpression

Human embryonic kidney (HEK293) cells were maintained in Dulbecco's modified eagle medium (DMEM, Gibco) containing 10% fetal bovine serum (FBS, HyClone), 1 mM sodium pyruvate and 1% penicillin/streptomycin solution (Gibco). HEK293 cells were transfected with GFP-TDP-43 containing full-length TDP-43 cDNA subcloned into pFUGW vector, by PolyJet™ DNA *in vitro* transfection reagent (Signagen Laboratories). Cells were seeded into a 6-well plate at a density of 5 × 10^5^ cells/well and incubated overnight. 2 μg of DNA was diluted to 100 μl with serum-free DMEM with high-glucose (4500 mg/L) (Invitrogen). In another tube, 6 μl of PolyJet reagent was diluted with 94 μl of serum-free DMEM with high-glucose. Diluted PolyJet reagent was added immediately into the diluted DNA solution and gently mixed by pipetting. The mixture was incubated for 15 minutes at room temperature to facilitate the formation of transfection complex and was added to 6-well plate containing HEK293 cells. After 24 h, 10 μM of MG-132 (Sigma-Aldrich) was treated for 0, 4, 8 and 12 h.

### Statistical analysis

All data are reported as the mean ± SEM. The statistical significance of differences between means was evaluated by two-tailed Student's *t* test. Differences were considered statistically significant at *p* < 0.05, as indicated by the asterisks: *P < 0.05, **P < 0.01 and ***P < 0.001.

## Supplementary Material

Supplementary figures.

## Abbreviations

FTLD: frontotemporal lobar degeneration
ALS: amyotrophic lateral sclerosis
HD: Huntington's disease
RBPs: RNA-binding proteins
mRNPs: messenger ribonucleoprotein particles
TDP-43: TAR DNA-binding protein 43
CNS: central nervous system
DIV: day *in vitro*
dSTORM: direct stochastic optical reconstruction microscopy
COMs: centers of mass
STAU2: Staufen 2
FMRP: Fragile X mental retardation protein
PBs: processing bodies
SGs: stress granules
Tg: transgenic
CaMKII: Ca^2+^/calmodulin-dependent kinase II
RNA-IP: RNA-immunoprecipitation
RPM: ribopuromycylation

## References

[1] Erkkinen MG, Kim MO, Geschwind MD (2018). Clinical Neurology and Epidemiology of the Major Neurodegenerative Diseases. Cold Spring Harb Perspect Biol.

[2] Skovronsky DM, Lee VM, Trojanowski JQ (2006). Neurodegenerative diseases: new concepts of pathogenesis and their therapeutic implications. Annu Rev Pathol.

[3] Wang J, Gu BJ, Masters CL, Wang YJ (2017). A systemic view of Alzheimer disease - insights from amyloid-beta metabolism beyond the brain. Nat Rev Neurol.

[4] Masters CL, Bateman R, Blennow K, Rowe CC, Sperling RA, Cummings JL (2015). Alzheimer's disease. Nat Rev Dis Primers.

[5] Murphy MP, LeVine H 3rd (2010). Alzheimer's disease and the amyloid-beta peptide. J Alzheimers Dis.

[6] Lashley T, Rohrer JD, Mead S, Revesz T (2015). Review: an update on clinical, genetic and pathological aspects of frontotemporal lobar degenerations. Neuropathol Appl Neurobiol.

[7] Warren JD, Rohrer JD, Rossor MN (2013). Clinical review. Frontotemporal dementia. Bmj.

[8] Ferrari R, Kapogiannis D, Huey ED, Momeni P (2011). FTD and ALS: a tale of two diseases. Curr Alzheimer Res.

[9] Bates GP, Dorsey R, Gusella JF, Hayden MR, Kay C, Leavitt BR (2015). Huntington disease. Nat Rev Dis Primers.

[10] Sweeney P, Park H, Baumann M, Dunlop J, Frydman J, Kopito R (2017). Protein misfolding in neurodegenerative diseases: implications and strategies. Transl Neurodegener.

[11] Ganguly G, Chakrabarti S, Chatterjee U, Saso L (2017). Proteinopathy, oxidative stress and mitochondrial dysfunction: cross talk in Alzheimer's disease and Parkinson's disease. Drug Des Devel Ther.

[12] Golde TE, Miller VM (2009). Proteinopathy-induced neuronal senescence: a hypothesis for brain failure in Alzheimer's and other neurodegenerative diseases. Alzheimers Res Ther.

[13] Golde TE, Borchelt DR, Giasson BI, Lewis J (2013). Thinking laterally about neurodegenerative proteinopathies. J Clin Invest.

[14] Conlon EG, Manley JL (2017). RNA-binding proteins in neurodegeneration: mechanisms in aggregate. Genes Dev.

[15] Cookson MR (2017). RNA-binding proteins implicated in neurodegenerative diseases. Wiley Interdiscip Rev RNA.

[16] Danner DJ RNA Binding Proteins: New Concepts in Gene Regulation: Am J Hum Genet. 2002; 71(5):1255.

[17] Buchan JR (2014). mRNP granules. Assembly, function, and connections with disease. RNA Biol.

[18] Liu EY, Cali CP, Lee EB (2017). RNA metabolism in neurodegenerative disease. Dis Model Mech.

[19] Batish M, van den Bogaard P, Kramer FR, Tyagi S (2012). Neuronal mRNAs travel singly into dendrites. Proc Natl Acad Sci U S A.

[20] Graber TE, Hebert-Seropian S, Khoutorsky A, David A, Yewdell JW, Lacaille JC (2013). Reactivation of stalled polyribosomes in synaptic plasticity. Proc Natl Acad Sci U S A.

[21] Krichevsky AM, Kosik KS (2001). Neuronal RNA granules: a link between RNA localization and stimulation-dependent translation. Neuron.

[22] Elvira G, Wasiak S, Blandford V, Tong XK, Serrano A, Fan X (2006). Characterization of an RNA granule from developing brain. Mol Cell Proteomics.

[23] Sephton CF, Cenik C, Kucukural A, Dammer EB, Cenik B, Han Y (2011). Identification of neuronal RNA targets of TDP-43-containing ribonucleoprotein complexes. J Biol Chem.

[24] Arai T, Hasegawa M, Akiyama H, Ikeda K, Nonaka T, Mori H (2006). TDP-43 is a component of ubiquitin-positive tau-negative inclusions in frontotemporal lobar degeneration and amyotrophic lateral sclerosis. Biochem Biophys Res Commun.

[25] Geser F, Martinez-Lage M, Robinson J, Uryu K, Neumann M, Brandmeir NJ (2009). Clinical and pathological continuum of multisystem TDP-43 proteinopathies. Arch Neurol.

[26] Tsai KJ, Yang CH, Fang YH, Cho KH, Chien WL, Wang WT (2010). Elevated expression of TDP-43 in the forebrain of mice is sufficient to cause neurological and pathological phenotypes mimicking FTLD-U. J Exp Med.

[27] Barmada SJ, Skibinski G, Korb E, Rao EJ, Wu JY, Finkbeiner S (2010). Cytoplasmic mislocalization of TDP-43 is toxic to neurons and enhanced by a mutation associated with familial amyotrophic lateral sclerosis. J Neurosci.

[28] Hanson KA, Kim SH, Tibbetts RS (2012). RNA-binding proteins in neurodegenerative disease: TDP-43 and beyond. Wiley Interdiscip Rev RNA.

[29] Narayanan RK, Mangelsdorf M, Panwar A, Butler TJ, Noakes PG, Wallace RH (2013). Identification of RNA bound to the TDP-43 ribonucleoprotein complex in the adult mouse brain. Amyotroph Lateral Scler Frontotemporal Degener.

[30] Alami NH, Smith RB, Carrasco MA, Williams LA, Winborn CS, Han SSW (2014). Axonal transport of TDP-43 mRNA granules is impaired by ALS-causing mutations. Neuron.

[31] Majumder P, Chu JF, Chatterjee B, Swamy KB, Shen CJ (2016). Co-regulation of mRNA translation by TDP-43 and Fragile X Syndrome protein FMRP. Acta Neuropathol.

[32] Lee EB, Lee VM, Trojanowski JQ (2011). Gains or losses: molecular mechanisms of TDP43-mediated neurodegeneration. Nat Rev Neurosci.

[33] Mackenzie IR, Rademakers R, Neumann M (2010). TDP-43 and FUS in amyotrophic lateral sclerosis and frontotemporal dementia. Lancet Neurol.

[34] Schoen M, Reichel JM, Demestre M, Putz S, Deshpande D, Proepper C (2015). Super-Resolution Microscopy Reveals Presynaptic Localization of the ALS/FTD Related Protein FUS in Hippocampal Neurons. Front Cell Neurosci.

[35] Dani A, Huang B, Bergan J, Dulac C, Zhuang X (2010). Superresolution imaging of chemical synapses in the brain. Neuron.

[36] Ishiguro A, Kimura N, Watanabe Y, Watanabe S, Ishihama A (2016). TDP-43 binds and transports G-quadruplex-containing mRNAs into neurites for local translation. Genes Cells.

[37] Liu-Yesucevitz L, Lin AY, Ebata A, Boon JY, Reid W, Xu YF (2014). ALS-linked mutations enlarge TDP-43-enriched neuronal RNA granules in the dendritic arbor. J Neurosci.

[38] Khalfallah Y, Kuta R, Grasmuck C, Prat A, Durham HD, Vande Velde C (2018). TDP-43 regulation of stress granule dynamics in neurodegenerative disease-relevant cell types. Sci Rep.

[39] Kiebler MA, Bassell GJ (2006). Neuronal RNA granules: movers and makers. Neuron.

[40] Khong A, Matheny T, Jain S, Mitchell SF, Wheeler JR, Parker R (2017). The Stress Granule Transcriptome Reveals Principles of mRNA Accumulation in Stress Granules. Mol Cell.

[41] Van Treeck B, Protter DSW, Matheny T, Khong A, Link CD, Parker R (2018). RNA self-assembly contributes to stress granule formation and defining the stress granule transcriptome. Proc Natl Acad Sci U S A.

[42] Bounedjah O, Desforges B, Wu TD, Pioche-Durieu C, Marco S, Hamon L (2014). Free mRNA in excess upon polysome dissociation is a scaffold for protein multimerization to form stress granules. Nucleic Acids Res.

[43] Sephton CF, Yu G (2015). The function of RNA-binding proteins at the synapse: implications for neurodegeneration. Cell Mol Life Sci.

[44] Palenzuela R, Gutierrez Y, Draffin JE, Lario A, Benoist M, Esteban JA (2017). MAP1B Light Chain Modulates Synaptic Transmission via AMPA Receptor Intracellular Trapping. J Neurosci.

[45] Lisman J, Schulman H, Cline H (2002). The molecular basis of CaMKII function in synaptic and behavioural memory. Nat Rev Neurosci.

[46] Lisman J, Yasuda R, Raghavachari S (2012). Mechanisms of CaMKII action in long-term potentiation. Nat Rev Neurosci.

[47] Moisse K, Volkening K, Leystra-Lantz C, Welch I, Hill T, Strong MJ (2009). Divergent patterns of cytosolic TDP-43 and neuronal progranulin expression following axotomy: implications for TDP-43 in the physiological response to neuronal injury. Brain Res.

[48] Scotter EL, Chen HJ, Shaw CE (2015). TDP-43 Proteinopathy and ALS: Insights into Disease Mechanisms and Therapeutic Targets. Neurotherapeutics.

[49] Qiu K, Du Y, Liu J, Guan JL, Chao H, Diao J (2020). Super-resolution observation of lysosomal dynamics with fluorescent gold nanoparticles. Theranostics.

[50] Feng H, Wang X, Xu Z, Zhang X, Gao Y (2018). Super-Resolution Fluorescence Microscopy for Single Cell Imaging. In: Gu J, Wang X, editors. Single Cell Biomedicine. Singapore: Springer Singapore.

[51] Lin F, Shelton SE, Espíndola D, Rojas JD, Pinton G, Dayton PA (2017). 3-D Ultrasound Localization Microscopy for Identifying Microvascular Morphology Features of Tumor Angiogenesis at a Resolution Beyond the Diffraction Limit of Conventional Ultrasound. Theranostics.

[52] Pandit R, Leinenga G, Götz J (2019). Repeated ultrasound treatment of tau transgenic mice clears neuronal tau by autophagy and improves behavioral functions. Theranostics.

[53] Cestra G, Rossi S, Di Salvio M, Cozzolino M (2017). Control of mRNA Translation in ALS Proteinopathy. Front Mol Neurosci.

[54] Fan AC, Leung AK (2016). RNA Granules and Diseases: A Case Study of Stress Granules in ALS and FTLD. Adv Exp Med Biol.

[55] Wu GY, Deisseroth K, Tsien RW (2001). Spaced stimuli stabilize MAPK pathway activation and its effects on dendritic morphology. Nat Neurosci.

[56] Wu CC, Lien CC, Hou WH, Chiang PM, Tsai KJ (2016). Gain of BDNF Function in Engrafted Neural Stem Cells Promotes the Therapeutic Potential for Alzheimer's Disease. Sci Rep.

[57] Herring BE, Nicoll RA (2016). Long-Term Potentiation: From CaMKII to AMPA Receptor Trafficking. Annu Rev Physiol.

[58] Tortosa E, Montenegro-Venegas C, Benoist M, Hartel S, Gonzalez-Billault C, Esteban JA (2011). Microtubule-associated protein 1B (MAP1B) is required for dendritic spine development and synaptic maturation. J Biol Chem.

[59] Djakovic SN, Schwarz LA, Barylko B, DeMartino GN, Patrick GN (2009). Regulation of the proteasome by neuronal activity and calcium/calmodulin-dependent protein kinase II. J Biol Chem.

[60] Conicella AE, Zerze GH, Mittal J, Fawzi NL (2016). ALS Mutations Disrupt Phase Separation Mediated by alpha-Helical Structure in the TDP-43 Low-Complexity C-Terminal Domain. Structure.

[61] Cook C, Zhang YJ, Xu YF, Dickson DW, Petrucelli L (2008). TDP-43 in neurodegenerative disorders. Expert Opin Biol Ther.

[62] Fang YS, Tsai KJ, Chang YJ, Kao P, Woods R, Kuo PH (2014). Full-length TDP-43 forms toxic amyloid oligomers that are present in frontotemporal lobar dementia-TDP patients. Nat Commun.

[63] Gong Y, Lippa CF, Zhu J, Lin Q, Rosso AL (2009). Disruption of glutamate receptors at Shank-postsynaptic platform in Alzheimer's disease. Brain Res.

[64] Majumder P, Chen YT, Bose JK, Wu CC, Cheng WC, Cheng SJ (2012). TDP-43 regulates the mammalian spinogenesis through translational repression of Rac1. Acta Neuropathol.

[65] Xu YF, Gendron TF, Zhang YJ, Lin WL, D'Alton S, Sheng H (2010). Wild-type human TDP-43 expression causes TDP-43 phosphorylation, mitochondrial aggregation, motor deficits, and early mortality in transgenic mice. J Neurosci.

[66] Wegorzewska I, Bell S, Cairns NJ, Miller TM, Baloh RH (2009). TDP-43 mutant transgenic mice develop features of ALS and frontotemporal lobar degeneration. Proc Natl Acad Sci U S A.

[67] Bai F, Witzmann FA (2007). Synaptosome proteomics. Subcell Biochem.

[68] Dempsey GT, Vaughan JC, Chen KH, Bates M, Zhuang X (2011). Evaluation of fluorophores for optimal performance in localization-based super-resolution imaging. Nat Methods.

[69] David A, Dolan BP, Hickman HD, Knowlton JJ, Clavarino G, Pierre P (2012). Nuclear translation visualized by ribosome-bound nascent chain puromycylation. J Cell Biol.

[70] Yasuda K, Zhang H, Loiselle D, Haystead T, Macara IG, Mili S (2013). The RNA-binding protein Fus directs translation of localized mRNAs in APC-RNP granules. J Cell Biol.

