# Supplementary material for: TDP-43 proteinopathy impairs mRNP granule mediated postsynaptic translation and mRNA metabolism
Source: Theranostics. 2021 Jan 1;11(1):330–45. doi: 10.7150/thno.51004 (PMC7681104; doi:10.7150/thno.51004)
Supplement: Supplementary file 1 — Supplementary figures. [file thnov11p0330s1.pdf]

Supplementary information

TDP-43 proteinopathy impairs mRNP granule mediated postsynaptic translation and mRNA metabolism

Chia-En Wong[1,2], Lee-Way Jin[3], Yuan-Ping Chu[2], Wei-Yen Wei[2], Pei-Chuan Ho[2] and Kuen-Jer Tsai[2,4*]

[1] Department of Medicine, College of Medicine, National Cheng Kung University, Tainan, Taiwan
[2] Institute of Clinical Medicine, College of Medicine, National Cheng Kung University, Tainan, Taiwan
[3] Department of Pathology and Laboratory Medicine, UC Davis Medical Center, California, USA
[4] Research Center of Clinical Medicine, National Cheng Kung University Hospital, College of Medicine, National Cheng Kung University, Tainan, Taiwan

Correspondence to: Kuen-Jer Tsai, Ph.D.
Institute of Clinical Medicine
College of Medicine
National Cheng Kung University
Tainan, Taiwan
Tel: +886-6-2353535-4254
Fax: +886-6-2758781
Email: kjtsai@mail.ncku.edu.tw

Supplementary Figure S2 is associated to Figure 2.
Supplementary Figure S3 is associated to Figure 7.
Supplementary Figure S4 is associated to Figure 8.

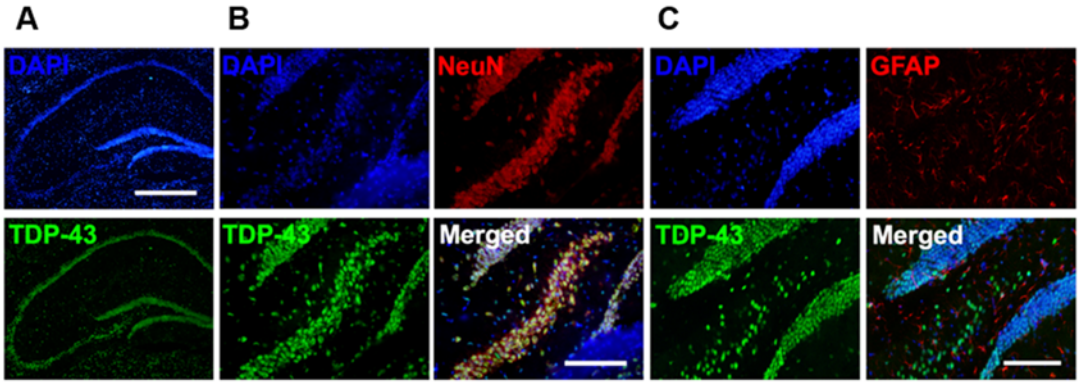

Supplementary Figure S1. The expression profile of TDP-43 protein in the mouse CNS. (A) The representative immunofluorescent images showed the distribution of TDP-43 in the mouse CNS, including the cortical layers and the hippocampus. Scale bar: 500 µm (B) The representative images showing the sharp-line signals in the hippocampal area indicated a predominantly somatic localization of TDP-43.by co-staining of TDP-43, neuron marker (NeuN) and nuclear marker (DAPI). Scale bar: 100 µm (C) Co-staining of TDP-43 and glial marker GFAP showing few colocalization.

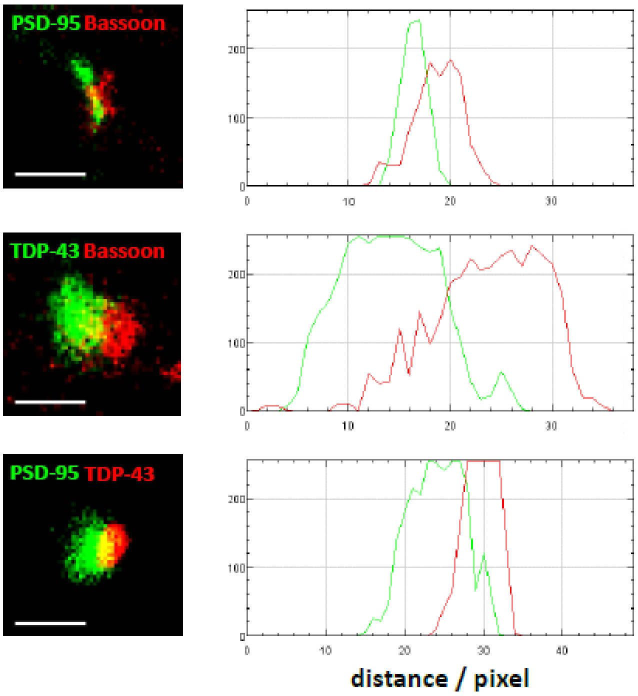

Supplementary Figure S2. The localization and measured distances of the respective protein pairs. The representative images and its corresponding histograms were displayed the position of COMs and distance of PSD-95, Bassoon and TDP-43 by calculated from intensity profiles along the transsynaptic axis. Scale bar: 400 nm

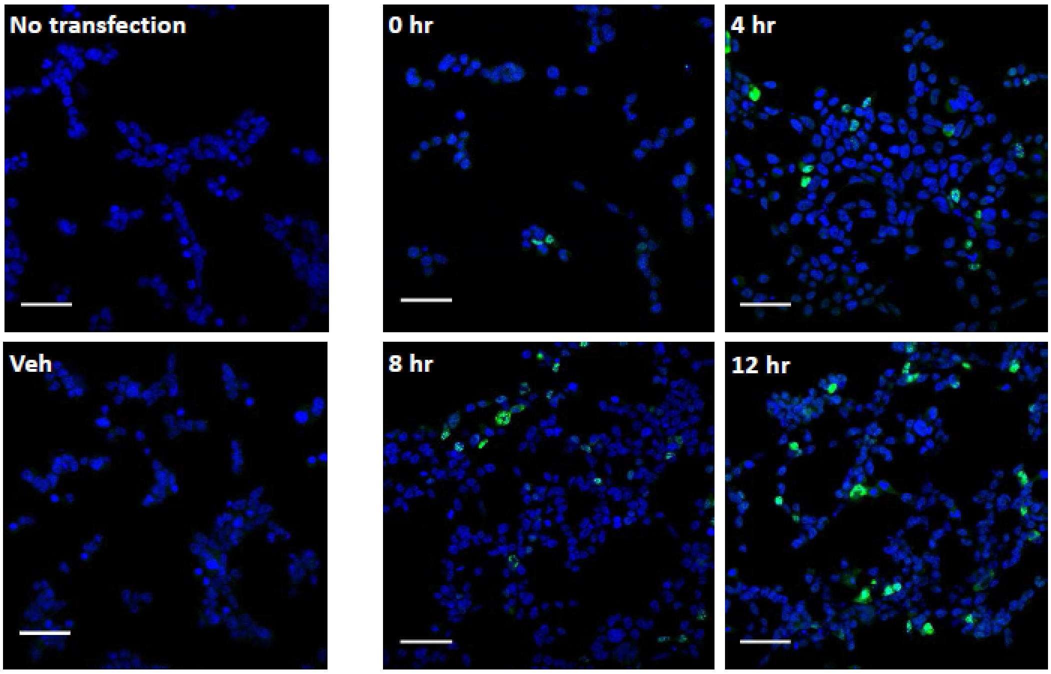

Supplementary Figure S3. The time-dependent formation of the pathological TDP-43 inclusions under proteasome inhibitor application. The HEK293T cells with TDP-43-GFP transfection were observed by confocal microscopy at 0, 4, 8 and 12 hours after MG-132 treatment. No transfection and TDP-43-GFP expression HEK 293T cells treated with vehicle (Veh) were served as control group, Scale bar: 100 μm

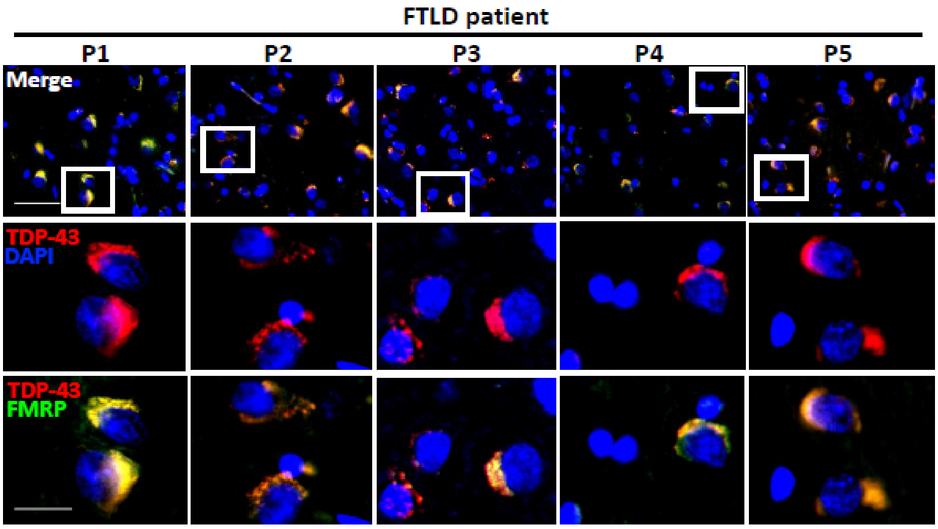

Supplementary Figure S4. The cerebral cortex images with TDP-43 and FMRP of all five FTLD-TDP patients. The immunostaining of neuronal mRNP granule marker, FMRP (green), and cytosolic TDP-43 aggregates (Red) were displayed high-degree colocalization (yellow). DAPI was served as nucleus signal. Scale bar: 50 µm and 10 µm (magnified images).
